# Investigation of the Surface Coating, Humidity Degradation, and Recovery of Perovskite Film Phase for Solar-Cell Applications

**DOI:** 10.3390/nano12173027

**Published:** 2022-08-31

**Authors:** Amal Bouich, Julia Marí-Guaita, Faisal Baig, Yousaf Hameed Khattak, Bernabé Marí Soucase, Pablo Palacios

**Affiliations:** 1 Escuela Técnica Superior de Ingeniería del Diseño, Universitat Politècnica de València, 46022 València, Spain; 2 Instituto de Energía Solar, ETSI Telecomunicación, Universidad Politécnica de Madrid, Ciudad Universitaria, s/n, 28040 Madrid, Spain; 3 Department Física Aplicada a las Ingenierías Aeronáutica y Naval, ETSI Aeronáutica y del Espacio, Universidad Politécnica de Madrid, Pz. Cardenal Cisneros, 3, 28040 Madrid, Spain; 4 Electrical Engineering Department, Federal Urdu University of Arts, Sciences and Technology, Islamabad 44000, Pakistan

**Keywords:** thin films, *APbI*_3_, organic/inorganic perovskite, optical properties, stability, SCAPS-1D, numerical analysis

## Abstract

Presently, we inquire about the organic/inorganic cation effect on different properties based on structure, morphology, and steadiness in preparing a one-step solution of APbI_3_ thin films, where A = MA, FA, Cs, using spin coating. This study was conducted to understand those properties well by incorporating device modeling using SCAPS-1D software and to upgrade their chemical composition. X-ray diffraction (XRD) was used to analyze the crystal structures. Atomic Force Microscopy (AFM) and Scanning Electron Microscopy (SEM) were conducted to characterize the surface morphology; photoluminescence, Transmission Electron Microscopy (TEM), and a UV–Visible spectrometer helped us to study the optical properties. The (110) plane is where we found the perovskite’s crystalline structure. According to the XRD results and by changing the type of cation, we influence stabilization and the growth of the $APbI_{3}$ absorber layer. Hither, a homogenous, smooth-surfaced, pinhole-free perovskite film and large grain size are results from the cesium cation. For the different cations, the band gap’s range, revealed by the optical analysis, is from 1.4 to 1.8 eV. Moreover, the stability of $CsPbI_{3}$ remains excellent for two weeks and in a ~60% humid environment. Based on the UV–Visible spectrometer and photoluminescence characterization, a numerical analysis for fabricated samples was also performed for stability analysis by modeling standard solar-cell structures $HTL/APbI_{3}/ETL$. Modeling findings are in good agreement with experimental results that $CsPbI_{3}$ is more stable, showing a loss % in PCE of 14.28%, which is smaller in comparison to $FAPbI_{3}$ (44.46%) and $MAPbI_{3}$ (20.24%).

## 1. Introduction

The decline of fossil fuels and global warming are responsible for the global demand for renewable energy resources and the development of advanced technology for producing them. The use of natural resources permits the production of energy from renewable energy resources [1,2]. It is imperative that the scientific community expands to make use of these resources efficiently. Global challenges of generating energy from renewable resources can be met with the help of solar energy [3,4]. Substantial efforts are required to develop novel photovoltaic technologies that guarantee cost reduction with enhanced efficiency. The research community has a rising interest in perovskite solar cells (PSCs) among other technologies because of the ease of the fabrication process and higher conversion efficiency [5,6,7,8,9,10].

The first PSC was reported in 2009 as having a power-conversion efficiency (PCE%) of 2.2% [11,12]. After extensive research, in 2011 researchers improved the efficiency by around 6.5% along with inadequate stability [13]. The conversion efficiency further improved to 9.7% in 2012 [14]. Researchers pay keen attention to PSCs because the PCE reached 15% in 2013 [15]. In 2014, Yalçin et al. presented PSC top efficiency of around 20% [16] and Devi et al. improves that PCE to 23.30% with 1.55 eV of bandgap in 2019 [17,18]. In the near future, the commercialization and stabilization of PSCs will increase greatly since 25.2% of efficient PSCs were recently verified and reported by KRICT and MIT. Results were also tested and verified by Newport PV Laboratory [19]. This is incredibly close to 26.7% efficient crystalline silicon solar cells [20,21]. The instant growth in the PSCs performance is the primary reason for the gigantic boost in the research, manufacturing, and development of PSCs. High absorption coefficient and long carrier diffusion length are also the cause for the further development of PSC technology [22,23].

Organic-inorganic halide perovskites are exceptionally fascinating absorber/active materials in thin-film technology due to their exceptional prominent device performance (solar cells and LEDs) and exceptional tunable optoelectronic properties [24,25,26,27,28]. Recently, a profound study has been made on hybrid perovskites $\left(APbX_{3}\right)$ due to their long carrier-diffusion length, high absorption, stability and carrier mobility, small effective hole/electron masses, and low exciting binding energies [29,30,31,32,33,34]. Consequently, the success of these synthesized compounds has been seen in manufacturing lasers [35,36] polarizers [37], diodes [38,39] photodetectors [40,41], and solar-cell [42,43] manufacturer technology.

Generally, the solar cells comprise the sandwiched configuration of having perovskite photoactive/absorbers type $ABX_{3}$, charge transport layers, and counter electrodes. Halide perovskite materials can be denoted by $ABX_{3}$; wherever A is an organic methylammonium $\left({\mathrm{CH}}_{3}{\mathrm{NH}}_{3}{}^{+}\;\mathrm{or}\;\mathrm{MA}\right)$ and formamidinium $\left({\mathrm{NH}}_{2}CH={\mathrm{NH}}_{2}{}^{+}\;or\;FA\right)$ cesium $\left({\mathrm{Cs}}^{+}\;\mathrm{or}\;\mathrm{Cs}\right)$ ions, B can be an inorganic cation $\left({\mathrm{Sn}}_{2}{}^{+}\;or{\;\mathrm{Pb}}_{2}{}^{+}\right),$ and X can be a halogen ion (${\mathrm{Cl}}^{-},\;{\mathrm{Br}}^{-}{\;\mathrm{or}\;I}^{-})$ [44,45,46]. Amongst them, the extremely conventional promising active materials are methylammonium lead iodide $({\mathrm{MAPbI}}_{3}$, formamidinium lead iodide $\left({\mathrm{FAPbI}}_{3}\right)$, and cesium lead iodide $\left({\mathrm{CsPbI}}_{3}\right)$ [47,48,49,50]. To realize the efficiency determination of PSCs, we should base the study on the interface of perovskite layers, the transportation process, and the charge extraction. Consequently, we can say that there is a parallel between each path of the crystal quality and the system’s efficiency, and at the interface, non-radiative recombination reduces. The development of the first leads to the enhancement of the other. In our work, the investigation primarily focuses on the cation lead iodide’s stability $APbI_{3}$ (where A can be Cs, MA, and FA), and the wide absorption range of the PSC phase. At UV–Vis wavelengths and to optimize photon absorption, the present studies concentrate on halide exchange to modify the bandgap. This work is divided into three main categories.

We describe a new method for altering the bandgap of halide perovskites by elaborating on cation materials. We have synthesized organic-inorganic lead halide perovskites ($APbI_{3}$, where A = mixed monovalent cation systems MA/Cs/FA), using the spin-coating process; this method is a low-cost technique for thin-film material deposition. After the successful fabrication of samples, we performed different characterization studies on as-deposited samples. Then we performed a detailed study of the degradation and recovery of the perovskite phase of deposited samples by studying their optical absorption and crystal structures along with the physical appearance of samples. Lastly, we performed a numerical analysis study of these materials to provide insight into physics for as-deposited, degraded, and recovered samples by simulating standard solar-cell structure $HTL/APbI_{3}/ETL$ in SCAPS-1D, where $APbI_{3}$ is replaced with $MAPbI_{3}$, $FAPbI_{3}$ and $CsPbI_{3}$.

## 2. Thin-Film Manufacture

### 2.1. Experimental Procedure

**Figure 1:**
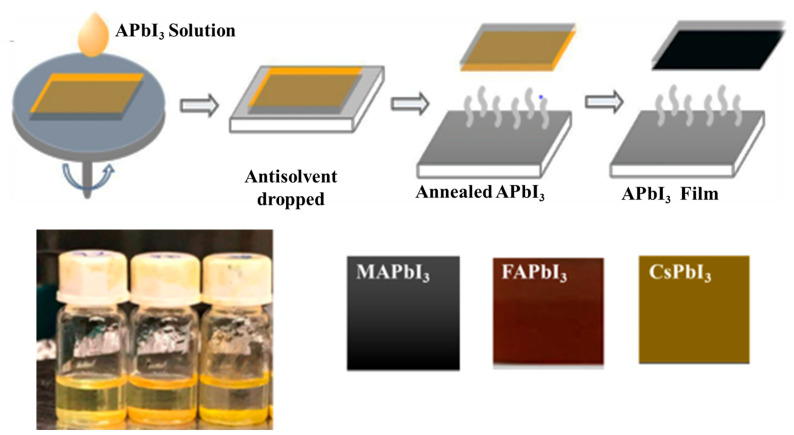
$APbI_{3}$ where A=MA/FA/Cs films were manufactured with a low-cost technique.

Lead (II) iodide ($PbI_{2}$), methylammonium iodide $\left(MAI\right)$, cesium iodide $\left(CsI\right)$, formidinium iodide $\left(FAI\right)$ purchased from sigma Aldrich, N,N-dimethylformamide anhydrous (DMF), and dimethyl sulfoxide (DMSO) from Thermo Scientific, the antisolvent chlorobenzene from Sigma-Aldrich, were used as precursor materials to fabricate the perovskite thin-film solutions. Then the prepared solution of $APbI_{3}$, where $\left(A=Cs,\;MA,\;FA\right),$ was spin-coated for 20 s at 2000 rpm on the FTO substrate. The deposition procedure is shown in Figure 1, where on top we displayed steps to deposit perovskite samples via the spin-coating method and at bottom of Figure 1 we displayed the as-deposited samples along with precursor solutions for perovskite materials.

### 2.2. Film Characterization

Different characterization techniques were used to evaluate the as-deposited samples of perovskite materials. The perovskite thin film’s crystal-structure analysis was performed by XRD RIGAKU Ultima IV diffractometer, SEM (Scanning Electron Microscopy) was performed to find the morphology of the deposited sample at different magnification levels, AFM (Atomic Force Microscopy) was performed to characterize the deposited film’s topography analysis, and TEM (Transmission Electron Microscopy) was also performed to authenticate the formation of perovskite structures. The absorption was calculated using a UV-Visible wavelength range of 300 to 850 nm, and photoluminescence (PL) was performed by He-Cd laser.

## 3. Results and Discussion

**Figure 2:**
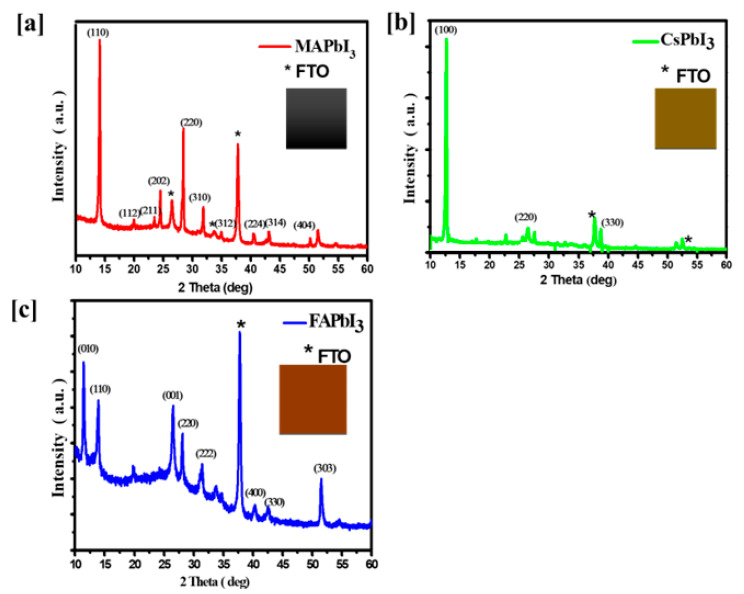
XRD patterns of (**a**) $MAPbI_{3}$, (**b**) $CsPbI_{3}$, and (**c**) $FAPbI_{3}$ thin films.

The impact of changing cation A on the thin films’ microstructure was explored by XRD Figure 2, where we can see the locations and the plans of diffractions peaks: 14.0 (110), 24.0 (202), 28.0 (220), 32.0 (222), 37.5 (400), and 52.0 (303). These crystal structures are fundamentally very similar; growth was shown at the peak at 2θ = 14°, which corresponds to $MAPbI_{3}$ shown in Figure 2a and $FAPbI_{3}$ (110) as (hkl) shown in Figure 2c. The orange phase is also for the (110) peak of $CsPbI_{3}$ shown in Figure 2b, which is the most prominent peak among the three compounds. However, a continuous displacement between the crystal structures is observed. Substantially, there is a highly crystalline phase, especially when there is an overly lattice strain. This remark shows the ability to substitute readily for the cations (MA, FA, and Cs) across the lattice without harming the crystal structure. The $MAPbI_{3}$ structure to be studied is correlated with the same diffraction peaks. Furthermore, the height (110) intensity is enhanced for the $CsPbI_{3}$ film.

The XRD pattern uncovered the enhanced crystallite orientation alongside the (110) plane. As a result of the solvent treatment, a tetragonal lattice has factored a = b = 8.919 Å and c = 11.920 Å, which corresponds to the space group I4/mcm, and the film of perovskite $MAPbI_{3}$ crystallizes. However, when $CsPbI_{3}$ was heat-treated for 10 min at 180 °C, these diffraction peaks can be allocated to cubic phase (a = 6.18 Å, space group Pm3m), and up to 180 °C in temperature, $a-CsPbI_{3}$ was formed as in the crystalline phases. These results indicated the efficiency of the synthesis of $CsPbI_{3}$ in the standard conditions, and the crystallization trend of perovskite was proper during synthesis [51].

The roughness and surface morphology changes with different cations in the perovskite thin films. The parameters are revealed in Table 1. Effective lattice strain has been calculated to know about the deformations of the grains in the surface of the film. To acquire the effective lattice strain (ɛ) Equation (1) was used [52].

(1) $$\beta \cos\left(\theta \right)=\frac{k\lambda }{D}+4\varepsilon \;\sin\left(\theta \right)$$

where λ is the wavelength of the X-ray, β is the full width half maximum (FWHM), k is a constant (0.94), and θ is the Bragg angle. Equation (2) was applied to determine the dislocation density of the crystal.

(2) $$\delta =\frac{1}{D^{2}}$$

**Figure 3:**
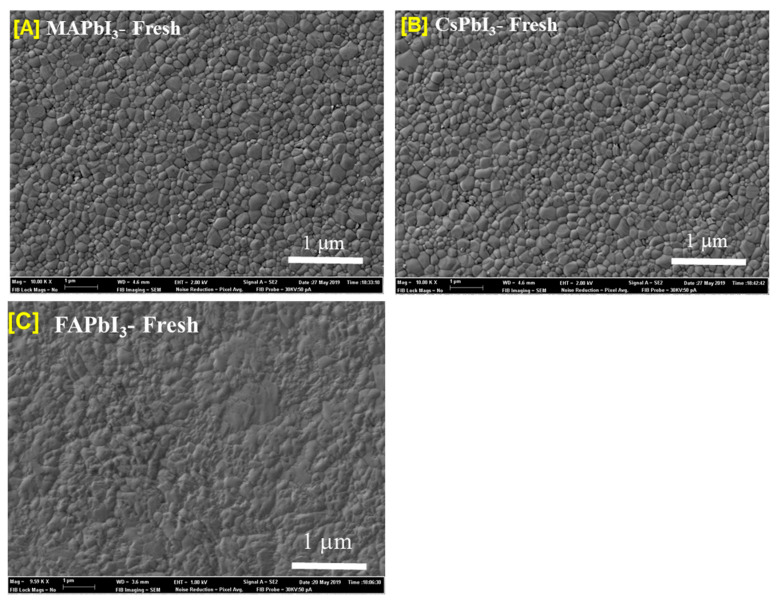
(SEM) images of the surface morphology of (**A**) $MAPbI_{3}$, (**B**) $CsPbI_{3}$, and (**C**) $FAPbI_{3}$.

Scanning electron micrography was the technique used to investigate the morphology of the films at many points in Figure 3. At first, large crystallites and a few large pinholes are the morphology of the $MAPbI_{3}$. As the change in the cation of MA by FA and Cs, there is a formation of a few crystals distributed randomly on the surface of $FAPbI_{3}$. The appearance of structures in destroyed shapes coincides with peaks corresponding to the yellow phase in XRD; this is the preferred crystal habit of the yellow $FAPbI_{3}$. Pinholes that are several nanometers were observed on the surface of $MAPbI_{3}$ annealed at 120 °C; in the case of $CsPbI_{3}$ perovskite, there are not plenty of pinholes in the thin film annealed at 180 °C. When the heat-treatment temperature was raised to 200 °C, it was clear that $CsPbI_{3}$ started to crystallize, and the grains were more regular. The effect could be explained by the $MAPbI_{3}$ perovskite becoming unstable under the same conditions, serving as a degraded model after a short amount of time. At the same time, the control film shows signs of $\delta -CsPbI_{3}$ at a temperature of 180 °C. To explain the better absorbance, there is a formation with a thicker and regular thickness of intimate contact with the underlying layer, which is compact and smooth with better-packed grains, which contained the resultant $CsPbI_{3}$ film annealed at 200 °C.

**Figure 4:**
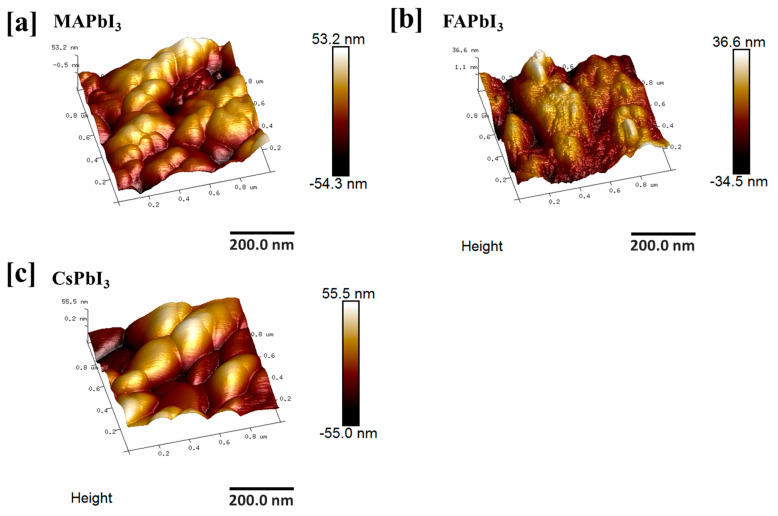
The topographic property of (**a**) $MAPbI_{3}$, (**b**) $FAPbI_{3}$, (**c**) $CsPbI_{3}$ films.

The surface morphology of samples is an important parameter for perovskite solar cells as they can directly affect the quantum efficiency (QE) of perovskite materials. To analyze the surface roughness of deposited perovskite material, AFM study was conducted, as the roughness parameter often results in many holes which create resistance and, consequently, decrease the charge mobility of carriers. Figure 4 shows the results for the surface roughness of perovskite materials, and from Figure 4 it is clear that surface height and valley point in MA- and Cs-doped perovskite are lesser than that of FA-doped perovskite material [53].

**Figure 5:**
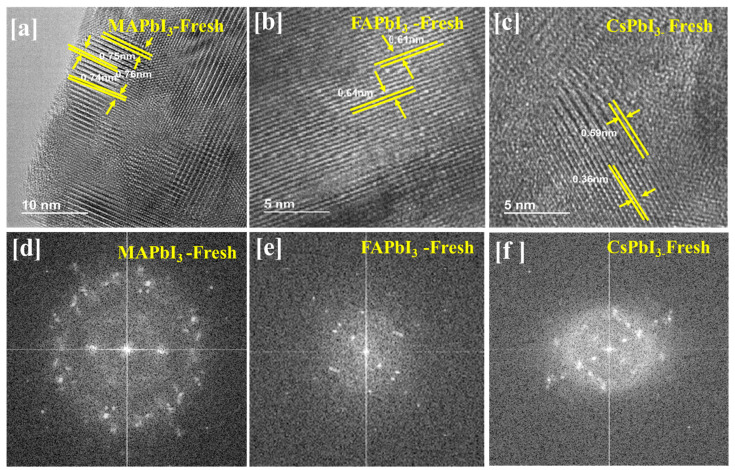
TEM images of the surface morphology of (**a**–**d**) $MAPbI_{3}$, (**b**–**e**) $FAPbI_{3}$, and (**c**–**f**) $CsPbI_{3}$.

Figure 5 indicates the TEM characterization of polycrystalline $MAPbI_{3}$ thin films. Further, 0.28 nm is the lattice fringe equivalent to (110) or (220) of the $MAPbI_{3}$ phase. $FAPbI_{3}$ thin films: 0.64 nm is the lattice fringe equivalent to (110) of the $FAPbI_{3}$ phase. $CsPbI_{3}$ thin films: 0.36 nm is the lattice fringe equivalent to (100) of the $CsPbI_{3}$ phase.

**Figure 6:**
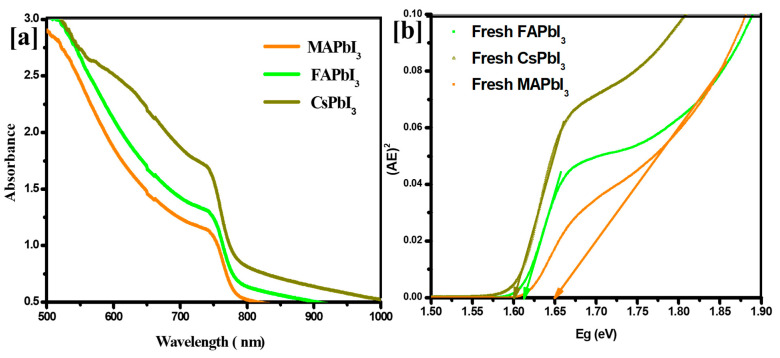
(**a**) Absorption and (**b**) Bandgap energy for of $MAPbI_{3}$, $FAPbI_{3}$, and $CsPbI_{3}$.

The PL measurements were canned at the ambient temperature as shown in Figure 6b. The PL peak intensity between 700–850 nm previously mentioned progressively increases with the $CsPbI_{3}$ film. However, by changing the cation A (FA) by MA and Cs, the PL intensities vary. A suggestion is that $CsPbI_{3}$ thin film is the optimal level at which it can better ambush, owing to the improvement of crystallinity and surface passivation, the absorption shift.

The corresponding UV–Vis spectra of $MAPbI_{3}$, $FAPbI_{3}$, and $CsPbI_{3}$ were recorded [300 nm–1000 nm] (Figure 6a). Different cations shift the absorption edge to a high wavelength, reducing the perovskite films’ defect density and increasing their crystallization quality. Furthermore, the optical bandgap is in good agreement.

**Figure 7:**
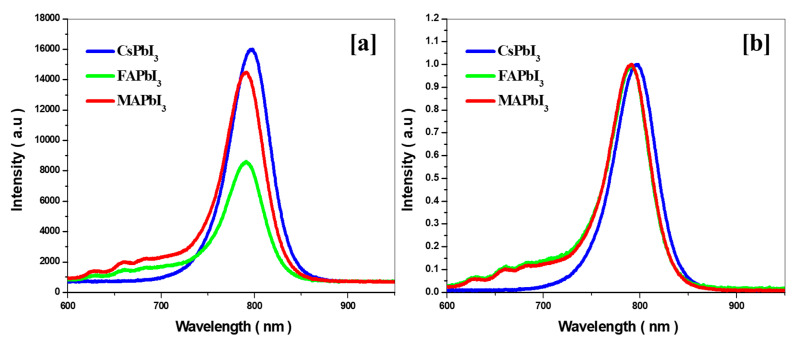
(**a**) Photoluminescence (PL) (**b**) normalized PL for $MAPbI_{3}$, $CsPbI_{3}$, and $FAPbI_{3}$ thin films.

The optical bandgap is in good agreement with the literature (Table 2), where it reduces drastically until an optimal level as shown in Figure 7. The intercalation of the cesium as a cation regulates the optical properties of $CsPbI_{3}$ semiconductor materials for photovoltaic devices [54].

## 4. Degradation Study

**Figure 8:**
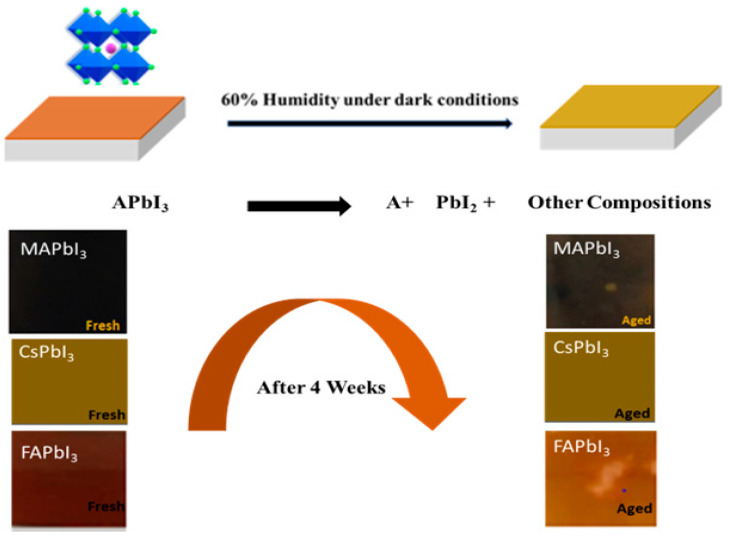
Degradation mechanism of $APbI_{3}$ in the air at 60% humidity and under dark conditions.

**Figure 9:**
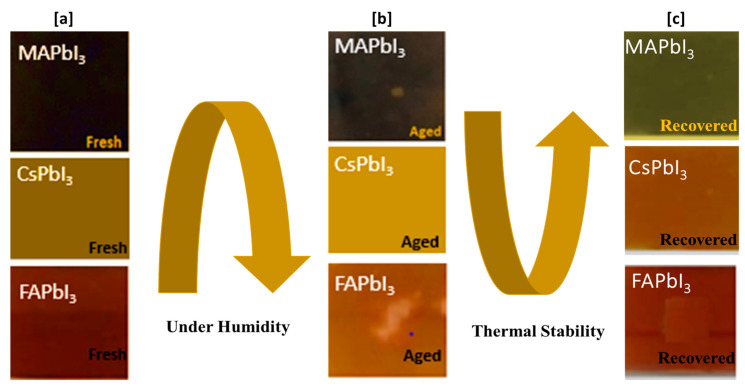
Recovery mechanism of $APbI_{3}$ in the air at 60% humidity and under dark conditions. (**a**) As deposited samples (**b**) Degradation of samples (**c**) Recovery after thermal treatment of samples.

The serious problems are the stability issues for the commercialization of perovskite solar cells. The halide perovskite components are related through weak interactions such as ionic and hydrogen bonding. The decomposition of organic species and the ion migration happen quickly in perovskite solar cells under moisture penetration as shown in Figure 8 and Figure 9 [55]. In this part, we discuss the stability from the viewpoint of cation exchange, $CsPbI_{3}$, $MAPbI_{3}$, and $FAPbI_{3}$ stabilization, and the best solution for reducing efficiency leakage. Figure 8 shows pictures of samples that were put in 60% humidity under dark conditions, and from Figure 8 it is clear that $MAPbI_{3}$ and $FAPbI_{3}$ have gone through degradation while $CsPbI_{3}$ shows resilience against humidity. A few pinholes were observed in samples just by visually inspecting the surface of the samples. The crystallography of the same samples was also analyzed by performing XRD of these samples.

**Figure 10:**
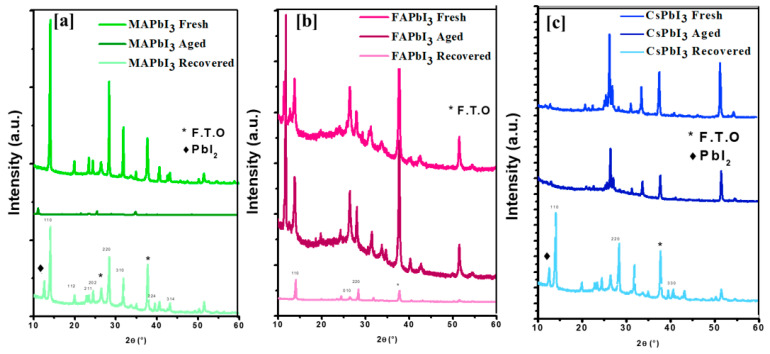
XRD characterization of $APbI_{3}$ samples in the air at 60% humidity and under dark conditions; (**a**) $MAPbI_{3}$, (**b**) $FAPbI_{3}$, (**c**) $CsPbI_{3}$.

**Figure 11:**
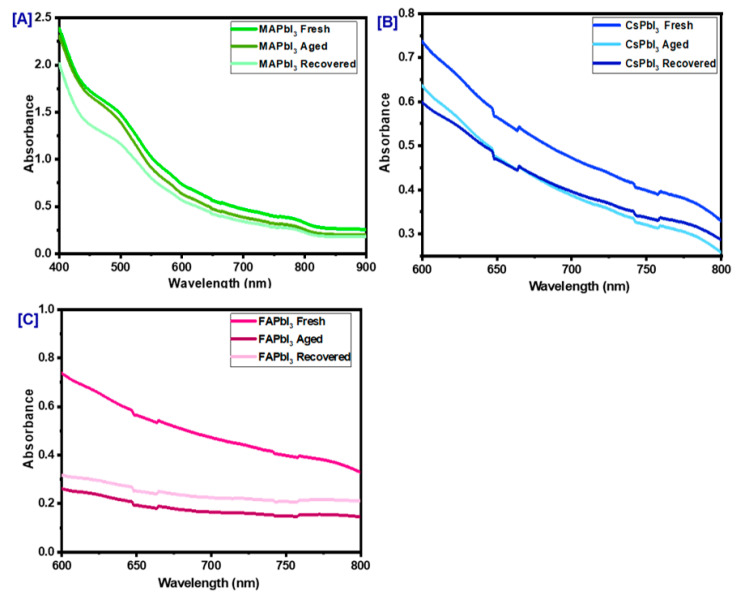
Optical absorption of $APbI_{3}$ samples in the air at 60% humidity and under dark conditions; (**A**) $MAPbI_{3}$, (**B**) $CsPbI_{3}$, (**C**) $FAPbI_{3}$.

Figure 10 shows the XRD patterns of fresh $MAPbI_{3}$, $FAPbI_{3}$, and $CsPbI_{3}$ thin films, aged for four weeks in the air at 60% humidity under dark conditions and recovered with thermal treatment under temperature 100 C. Although no technique is generally used to measure the stability of perovskite thin films, a simple procedure was developed to analyze them. By comparing the values of the intensities, this method was developed to detect the most stable thin film. Relative to fresh spectra, essential changes do not happen in ranges; for example, in the appearance of new peaks, a percentage decrease in intensity appears to relate with stability since the number of perovskite diffraction planes is proportional to the total power. This phenomenon explains that the new the peaks that appear on older films correspond to new phases and demonstrate the partial degradation of the $MAPbI_{3}$, $FAPbI_{3}$, and $CsPbI_{3}$ thin films. The recovered $FAPbI_{3}$ and $CsPbI_{3}$ samples showed significantly increased stability by thermal treatment due to the hysteretic effect of these materials related to phase transition. Remarkably, the intensity of the recovered pieces is higher than the aged $CsPbI_{3}$ and $FAPbI_{3}$ prepared. On the other hand, the $MAPbI_{3}$ film showed low stability compared to the perovskite based on cesium, see Figure 10. This study suggests that $CsPbI_{3}$ improves crystal quality and has high stability. However, we can see the appearance of the non-perovskite Ɣ phase for the $FAPbI_{3}$ film in the spectrum in Figure 10b, showing that parts of the structure had deteriorated in new phases; thus, we can announce that this study proves that $CsPbI_{3}$ is the most efficient in these conditions. The absorption results confirm this conclusion where the aged $CsPbI_{3}$ and $FAPbI_{3}\;and$ $MAPbI_{3}$ samples show a dramatic decrease compared to the fresh and recovered samples, and these results are plotted in Figure 11.

## 5. Film Recovering

**Figure 12:**
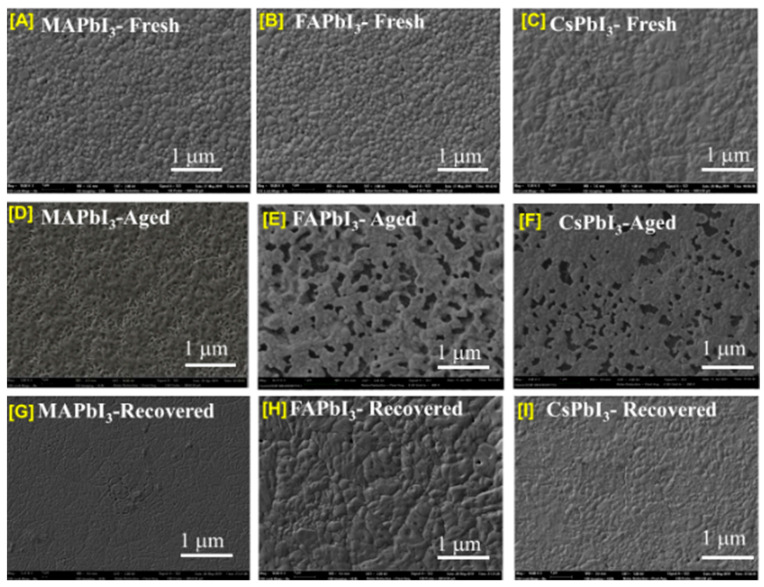
Fresh, degraded and recovered samples of $APbI_{3}$ in the air at 60% humidity and under dark conditions: (**A**,**D**,**G**) $MAPbI_{3}$ (**B**,**E**,**H**) $FAPbI_{3}$ (**C**,**F**,**I**) $CsPbI_{3}$.

From the photographs $\mathrm{APb}I_{3}$, as shown in Figure 8 and Figure 9, the color changes from a deep color to a clear color due to the dissociation or phase transition of perovskite. The same process was also analyzed with the subject to SEM studies by analyzing the surfaces of the deposited, degraded, and thermally treated samples. From Figure 12, a pinhole and change in surface morphology are apparent for pure aged FA and MA samples compared to fresh ones, but we can note that for Cs samples that show good stability and are well recovered by thermal treatment, the surface shows fewer pinholes. This is in agreement with the results of the absorption spectra (plotted in Figure 11) and visual inspection in Figure 9, which confirms the stability; here, we can offer the route to enhance the stability of $APbI_{3}$. We confirm that the incorporation of $\left(Cs\right)$ enhances the stabilization of perovskite and our results are in good agreement with the literature [56].

## 6. Numerical Analysis

Device modeling for as-deposited, aged, and recovered samples was performed in SCAPS, and the simulation parameters that were used for the given device structure $HTL/APbI_{3}/ETL$ are given in Table 3, Table 4 and Table 5.

SCAPS-1D is a solar-cell capacitance software that is used to calculate the functional parameters of the solar cell, such as short circuit current (Jsc), open-circuit voltage (Voc), Fill factor (FF%), and power-conversion efficiency (PCE%), based on the input parameters given in Table 3 and Table 4 and the absorption profile for each layer [57,58]. SACPS-1D, over years, has proven to be a significant tool for understanding the physics of solar cells and it has been comprehensively used for perovskite solar cells [59,60,61,62,63,64]. SCAPS also calculates AC quantities, electron/hole densities, quantum efficiencies (QE%)/spectral response, total recombination currents, energy band diagrams, and current density vs. voltage characteristics [65,66,67,68,69,70,71,72,73] It is based on drift-diffusion differential equations and Poisson’s carrier (electron/hole transport) continuity equations [63,64].

The $HTL/APbI_{3}/ETL$ desired structures were simulated in SCAPS-1D software; in this, Spiro−OMeTAD was used as HTL, $APbI_{3}$ is a perovskite absorber layer with A replacing $\left(MA,FA,Cs\right)$ and $TiO_{2}$ is used as ETL layer. The physical parameters for all structures remained constant, as shown in Table 3 and Table 4, and the only things that we changed in the absorption profile for the absorber layer were “as-deposited”, “after-degradation” and “recovery”, along with the band gap of the absorber layers.

- JV and QE% as deposited.
- JV and QE% after degradation.
- JV and QE% after recovery.

**Figure 13:**
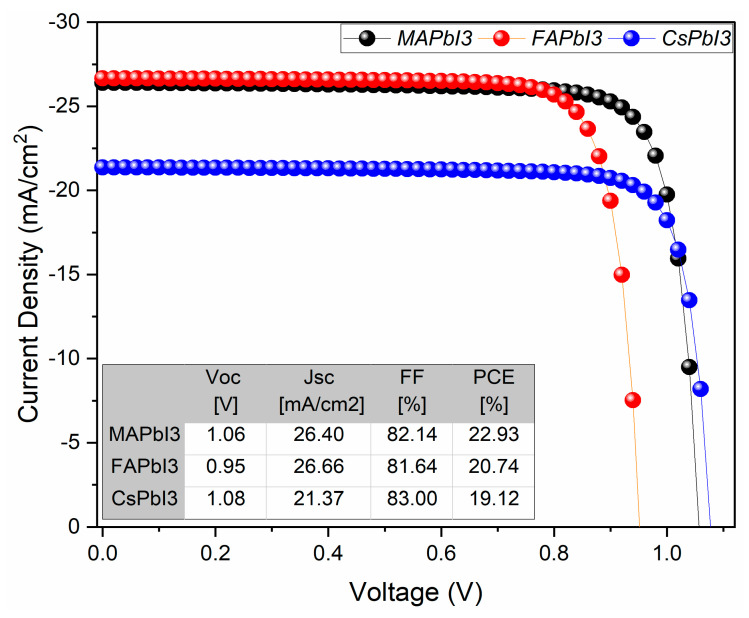
J-V curve of fabricated samples; $MAPbI_{3}$, $FAPbI_{3}$, and $CsPbI_{3}$.

**Figure 14:**
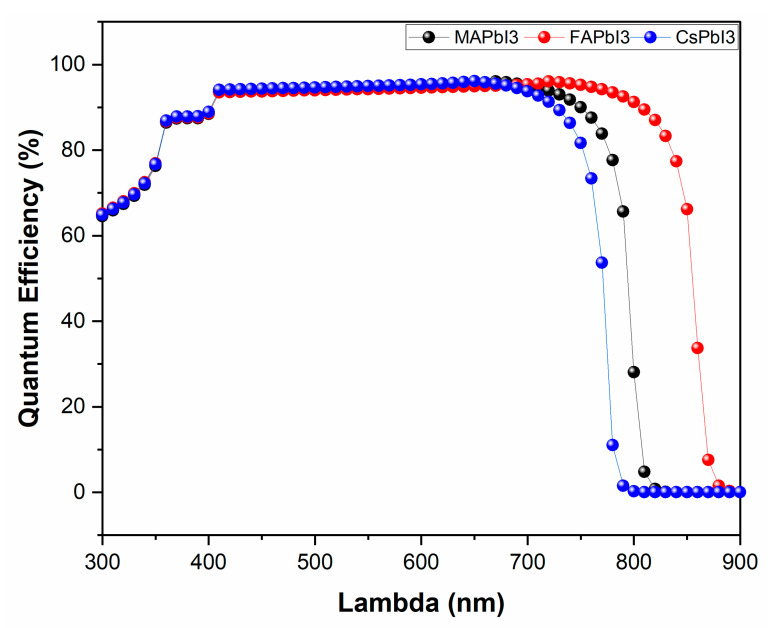
Quantum efficiency of fabricated samples; $MAPbI_{3}$, $FAPbI_{3}$, and $CsPbI_{3}$.

The results for JV characteristics along with QE% for as-deposited samples were plotted in Figure 13 and Figure 14. From the figures, it is visible that solar-cell structure with $MAPbI_{3}$ as the absorber layer has a higher PCE% than other structures, as well as QE%. The reason for this is because of good band alignment and high absorption coefficient with respect to the other two samples, whereas high open-circuit voltage was achieved for the sample $CsPbI_{3}$ due to its wider band gap.

**Figure 15:**
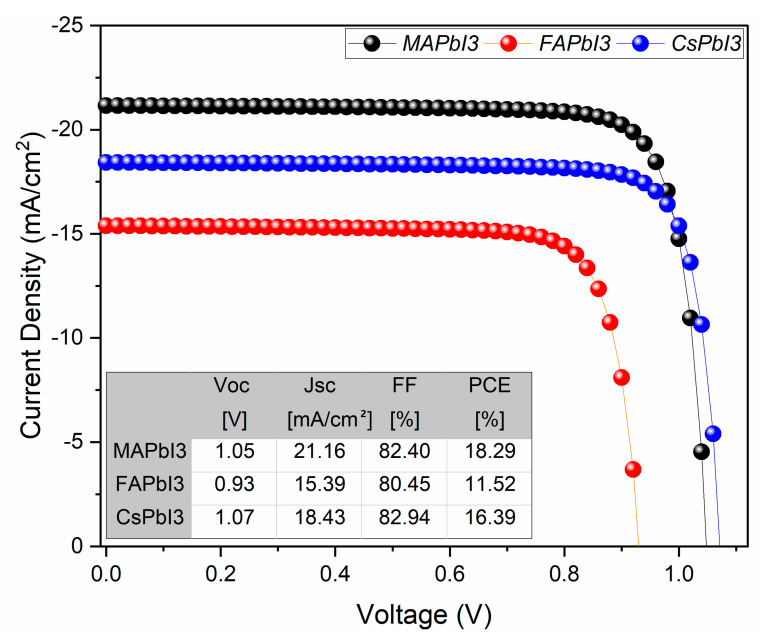
J-V curve of fabricated samples degraded; $MAPbI_{3}$, $FAPbI_{3}$, and $CsPbI_{3}$.

**Figure 16:**
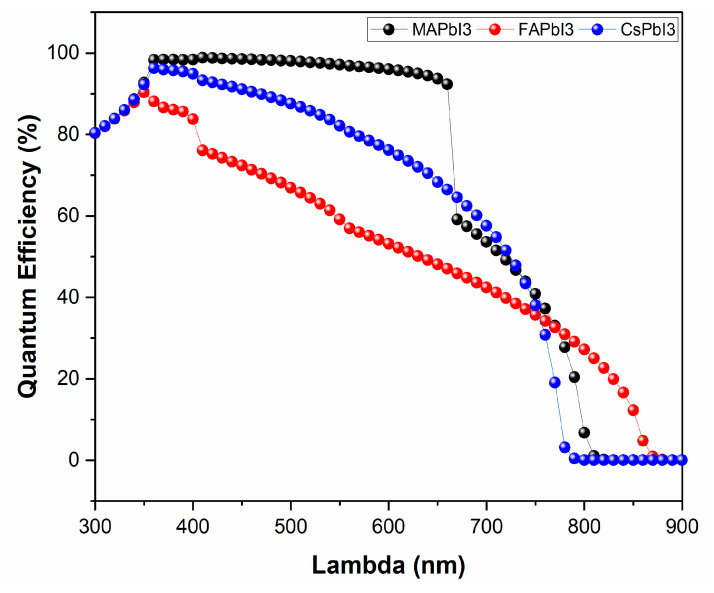
Quantum efficiency of fabricated samples degraded; $MAPbI_{3}$, $FAPbI_{3}$, and $CsPbI_{3}$.

Similarly, results for JV and QE characteristics after degradation were also plotted in Figure 15 and Figure 16 below. Figure 15 clearly shows that degradation highly affects the performance of $FAPbI_{3}$ by reducing its open-circuit voltage ($V_{oc}$) and short circuit current ($J_{sc}$).

Based on these results we calculated the percentage loss of PCE in each solar cell, and for this, a formula was devised to find the percentage of loss in PCE% during the degradation of a solar cell. *% loss* formula is shown in Equation (3) below

(3) $$\%\;loss=\frac{PCE_{FS}-PCE_{DS}}{\left|PCE_{FS}\right|}\times 100$$

where $PCE_{FS}$ is the fabricated samples power-conversion efficiency estimated in SCAPS-1D software and $PCE_{DS}$ is the degraded samples power-conversion efficiency estimated in SCAPS-1D after degradation. Results for *% loss* are shown in Table 5, and it is clearly shown that $FAPbI_{3}$ is highly affected by degradation and $CsPbI_{3}$ shows more stability.

**Figure 17:**
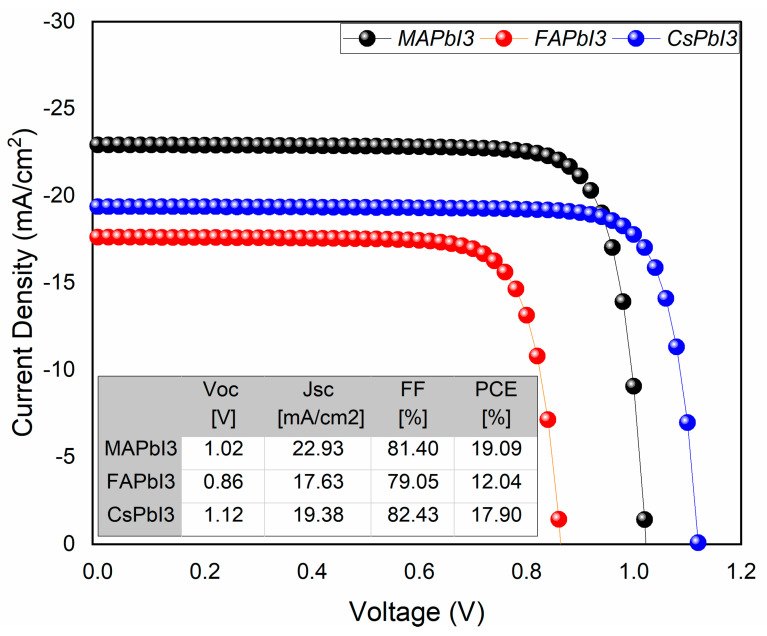
J-V curve of fabricated samples recovered; $MAPbI_{3}$, $FAPbI_{3}$, and $CsPbI_{3}$.

**Figure 18:**
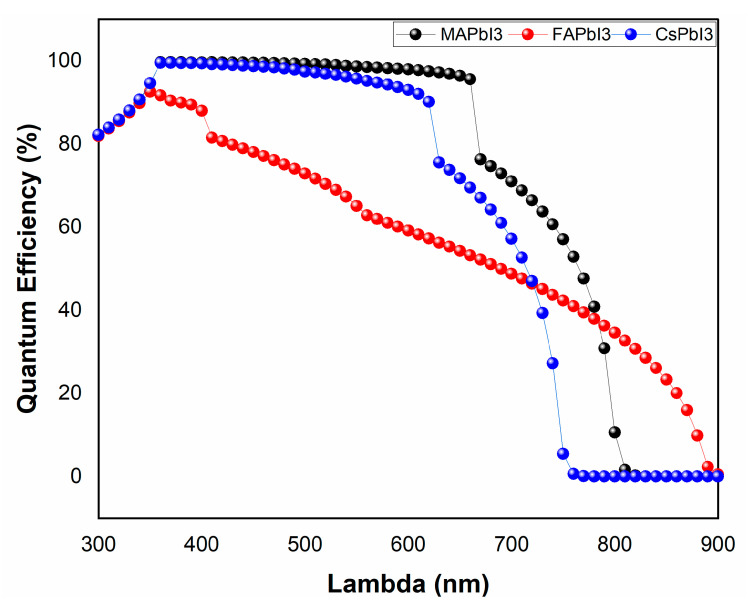
Quantum efficiency of fabricated samples recovered; $MAPbI_{3}$, $FAPbI_{3}$, and $CsPbI_{3}$.

Similarly, in the last section, we apply the recovery mechanism in SCAPS-1D for given solar-cell structures, and results are drawn in Figure 17 and Figure 18.

Similar to *% loss*, a formula was also devised to find the *% gain* after recovery of perovskite thin films, and the formula is shown below in Equation (4).

(4) $$\%\;gain=\frac{PCE_{RS}-PCE_{DS}}{\left|PCE_{DS}\right|}\times 100$$

The results for the given formula are drawn in Table 6. Where $PCE_{RS}$ is the recovered samples power-conversion efficiency and $PCE_{DS}$ is the degraded samples power-conversion efficiency. From Table 6, it is clearly shown that $CsPbI_{3}$ has proven to be more stable.

## 7. Conclusions

The one-step spin-coating technique has successfully prepared $CsPbI_{3}$ thin films. The effect of cation A was investigated by XRD, SEM, optical analysis, and SCAPS-1D solar-cell numerical analysis software. The XRD analysis displays an extraordinary intensity of peak (110) by treating $CsPbI_{3}$ film with the chlorobenzene antisolvent, leading to large grains of $CsPbI_{3}$ thin film examined by SEM and AFM analysis. Furthermore, the enhancement of light absorption was observed more effectively. To investigate the degradation effect on device performance, a numerical analysis was performed with device structures $Spiro-OMeTAD/MAPbI_{3}/TiO_{2}$, $Spiro-OMeTAD/FAPbI_{3}/TiO_{2}$ and $Spiro-OMeTAD/CsPbI_{3}/TiO_{2}$. Based on the results presented, it was found that $CsPbI_{3}$ thin films are suitable candidates for efficient, stable, and durable perovskite devices.

## Figures and Tables

**Table 1:** $APbI_{3}$ thin films XRD parameters.

Sample ID	Lattice Strain $\left(\varepsilon \times 10^{-3}\right)$	Grain Size $\left(nm\right)$	Dislocation Density $\left(nm^{-1}\right)$	Roughness $\left(nm\right)$
** $MAPbI_{3}$ **	9.05	331	$0.91\times 10^{-5}$	145
** $CsPbI_{3}$ **	8.71	345	$0.84\times 10^{-5}$	420
** $FAPbI_{3}$ **	8.13	283	$1.13\times 10^{-5}$	231

**Table 2:** $APbI_{3}$ thin films’ optical properties.

Sample ID	Optical Band Gap by Absorption		Emission PL Peak		Stokes Shift
	$\lambda c\;\left(nm\right)$	$Eg\;\left(eV\right)$	$\lambda \;\left(nm\right)$	$Eg\;\left(eV\right)$	$\left(meV\right)$
** $MAPbI_{3}$ **	719	1.55	760	1.40	150
** $FAPbI_{3}$ **	795	1.50	770	1.35	150
** $CsPbI_{3}$ **	752	1.53	768	1.36	180

**Table 3:** Simulated-device physical input parameters [58,70,71,72,73].

Parameters	Spiro−OMeTAD (HTL)	$MAPbI_{3}\;(\mathrm{Absorber})$	$FAPbI_{3}\;(\mathrm{Absorber})$	$CsPbI_{3}\;(\mathrm{Absorber})$	$TiO_{2}\;(\mathrm{ETL})$
$W\;\left(µm\right)$	0.6	0.4	0.4	0.4	0.1
$E_{g}$ $\left(eV\right)$	3	1.55	1.5	1.53	3.2
χ $\left(eV\right)$	2.45	3.9	4.0	3.88	4
$\in_{r}$	3	10	6.6	6	9
$N_{c}\left(cm^{-3}\right)$	$2.2\times 10^{18}$	$2.8\times 10^{18}$	$1.2\times 10^{19}$	$1.1\times 10^{20}$	$1\times 10^{19}$
$N_{v}$ $\left(cm^{-3}\right)$	$1.1\times 10^{19}$	$3.9\times 10^{18}$	$2.9\times 10^{18}$	$8\times 10^{19}$	$1\times 10^{19}$
n, p $\left(cm^{-3}\right)$	$1\times 10^{15}$	$1\times 10^{14}$	$1\times 10^{14}$	$1\times 10^{14}$	$1\times 10^{16}$
$\mu_{e}\;\left(cm^{2}/Vs\right)$	0.0002	11.8	2.7	16	20
$\mu_{p}$ $\left(cm^{2}/Vs\right)$	0.0002	11.8	2.8	16	10

**Table 4:** Defects in layers and interfaces.

Defect Properties	Spiro−OMeTAD	Absorber	Spiro−OMeTAD/ Absorber
$N_{t}$	$1\times 10^{16}\;\left({\mathrm{cm}}^{-3}\right)$	$3\times 10^{14}\;\left({\mathrm{cm}}^{-3}\right)$	$1\times 10^{14}\;\left({\mathrm{cm}}^{-2}\right)$
$E\;\left(eV\right)$	0.6	0.6	0.32
$\delta_{e}\left(cm^{2}\right)$	$1\times 10^{-14}$	$1\times 10^{-14}$	$1\times 10^{-16}$
$\delta_{h}\left(cm^{2}\right)$	$1\times 10^{-14}$	$1\times 10^{-14}$	$1\times 10^{-16}$
$N_{t}$ : Total density E : Energy level $\delta_{h}\;,\delta_{e}$ : Holes and electrons capture cross section area			

**Table 5:** Percentage loss in different fabricated solar cells PCE; $MAPbI_{3}$, $FAPbI_{3}$, and $CsPbI_{3}$.

Samples	Fabricated Samples PCE [%]	Degraded Samples PCE [%]	Loss Percentage in PCE [%]
$MAPbI_{3}$	22.93	18.29	20.24
$FAPbI_{3}$	20.74	11.52	44.46
$CsPbI_{3}$	19.12	16.39	14.28

**Table 6:** Percentage gain in different fabricated solar cells PCE; $MAPbI_{3}$, $FAPbI_{3}$, and $CsPbI_{3}$.

Samples	Degraded Samples PCE [%]	Recovered Samples PCE [%]	Gain Percentage in PCE [%]
$MAPbI_{3}$	18.29	19.09	4.37
$FAPbI_{3}$	11.52	12.04	4.51
$CsPbI_{3}$	16.39	17.90	9.21

## Data Availability

All data is included in the manuscript.
